# miR-29a-3p/T-bet Regulatory Circuit Is Altered in T Cells of Patients With Hashimoto’s Thyroiditis

**DOI:** 10.3389/fendo.2018.00264

**Published:** 2018-05-24

**Authors:** Stana Tokić, Mario Štefanić, Ljubica Glavaš-Obrovac, Amit Kishore, Zdenka Navratilova, Martin Petrek

**Affiliations:** ^1^Department of Medical Chemistry, Biochemistry and Clinical Chemistry, Faculty of Medicine, University of Osijek, Osijek, Croatia; ^2^Department of Pathological Physiology, Palacky University and Faculty Hospital, Olomouc, Czechia; ^3^Department of Nuclear Medicine and Oncology, Faculty of Medicine, University of Osijek, Osijek, Croatia

**Keywords:** Hashimoto disease, T-lymphocytes, hsa-miR-29a, hsa-miR-210, hsa-miR-9, disease attributes, thyroid gland

## Abstract

**Objective:**

Hashimoto’s thyroiditis (HT) is a common autoimmune thyroid disorder that frequently evolves from asymptomatic, T-cell mediated chronic inflammation toward overt hypothyroidism. Previously, we have demonstrated a role for T-bet, a T helper 1/CD8^+^ T cell transcription factor (TF), and FoxP3, a regulatory T cell TF, in disease progression and severity, but the basis behind their altered mRNA expression remains unknown. In this study, we aimed to leverage the role for microRNAs, representing negative transcriptional regulators, across the spectrum of HT clinical presentations using the same, well-characterized RNA sample cohort.

**Method:**

Ten hypothyroid, untreated patients (hypoHT), 10 hypothyroid cases rendered euthyroid by l-thyroxine therapy (substHT), 11 spontaneously euthyroid HT subjects (euHT), and 10 healthy controls (ctrl) were probed for three candidate immunoregulatory miRNA (miR-9-5p, miR-29a-3p, and miR-210-3p) using quantitative real-time PCR measurements. Data were normalized to U6snRNA and fold difference in expression calculated by the efficiency corrected 2^−ΔΔCt^ model.

**Results:**

Compared to healthy controls, peripheral blood (PB) T cells of HT patients exhibited significantly diminished miR-29a-3p expression levels [median expression levels (IQR), HT vs CTRL, 0.62 (0.44–1.01) vs 1.373 (0.63–2.7), *P* = 0.046], and a similar, but not significant decline in miR-210-3p abundance [HT vs CTRL, 0.64 (0.39–1.31) vs 1.2 (0.5–2.56), *P* = 0.24, Wilcoxon test]. A significant inverse correlation was observed between the two differentially expressed transcripts, T-bet mRNA and miR-29a-3p. Moreover, altered miR-29a-3p/T-bet expression in T cells of untreated HT patients was related to low serum FT4, high serum thyrotropin, and decreased thyroid volumes. Of note, miR-210-3p expression was positively correlated to HIF1α, and inversely to FoxP3 mRNA levels, but no evidence of differential expression for any of these miRNA–mRNA pairs was observed. Finally, miR-9-5p expression levels were no different in HT vs control comparisons, or related to clinicopathological features.

**Conclusion:**

T cell miR-29a-3p is downregulated in HT patients and associated with clinical and biochemical parameters of progressive thyroid injury, plausibly subsequent to altered control of T-bet expression in PB T cells. As such miR-29a-3p/T-bet axis should be further explored as a biomarker or as a plausible target for therapeutic interventions in HT.

## Introduction

Hashimoto’s thyroiditis (HT) is a multifactorial, autoimmune disorder characterized by the presence of thyroid-specific autoantibodies, chronic lymphocytic infiltration of the thyroid gland, and, eventually, hypothyroidism (1). Pathologically, HT is mediated by aberrant T helper type 1 (Th1), Th17, Treg, and cytotoxic (Tc) CD8^+^ responses, which typically require master regulator transcription factors (TFs) T-bet, RORγt, FOXP3, and EOMES, respectively, for their development. Of these, T-bet (2, 3), RORγt (4–6), and FOXP3 (3, 7, 8) have all been implicated in the ontogeny and severity of HT in humans; however, the mechanisms beyond the reported deregulated expression of these TFs in patients’ T cells remain unclear.

Certain microRNAs (miRNAs, miR) act by negatively regulating the expression of key master regulators within this network (9). miRNAs are a class of single-stranded 19–25 nucleotide long non-coding RNAs that bind 3′-untranslated region of target mRNA and result in either translation inhibition or mRNA degradation. Among these, miR-29a-3p limits Th1/Tc1 bias by targeting interferon (IFN)-γ-inducing TFs EOMES and T-bet (10). In Treg cells, miR-9 and miR-210 are markedly downregulated in CD4^+^CD25^+^CD127^low^ (11) and CD8^+^CD25^+^ (12) subsets compared to non-Treg T cells. By contrast, miR-210, the hypoxia-induced miR, is greatly enhanced in activated, effector CD8^+^ and CD4^+^ T cells under Treg- > Th17-polarizing conditions (13), whereby it inhibits FOXP3 expression and impairs the immunosuppressive functions of CD4^+^ Treg cells. HIF1α, a TF-controlling Th17 polarization and CD8^+^ effector differentiation (14), is both an upstream miR-210 regulator and a miR-210 target, thus; miR-29, miR-210, and miR-9, all seem to affect a broad spectrum of differentiation pathways, including those responsible for producing effector and regulatory cells. Nevertheless, despite evidence supporting the importance of these miRs in controlling disease-associated TFs, their respective regulatory networks in the context of HT, disease severity, and clinical presentation remain mostly unexplored.

We previously described increments in transcript levels of Th1 and Treg-associated, T-bet, and FOXP3, but not Th17-related HIF1α TF, in peripheral blood (PB) T cells of severely affected HT patients. In this study, the same RNA sample cohort was probed for expression levels of their respective miRNA pairs. Thus, we assessed the transcriptional patterns of miR-29-3p, miR-210-3p, and miR-9-5p in bulk PB T cells and attempted to combine the selected TFs and miRNAs to study their co-regulatory networks, and effects on clinical features of HT patients with distinct patterns of disease severity.

## Materials and Methods

### Subjects

In this study, we built upon our previous work by screening for selected miRNAs in an established and well-characterized cohort of HT cases and healthy controls who had been previously typed for *T-bet, FoxP3, BLIMP1*, and *HIF1*α expression in bulk PB T cells. HT was classified as (1) hypothyroid, untreated [hypoHT, *n* = 10, 2 males], (2) spontaneously euthyroid HT [euHT, *n* = 12, all females], and (3) rendered euthyroid by hormone replacement therapy [substHT, *n* = 10, 1 male, median l-thyroxine (T4) dose 1.13 µg/kg body mass daily, median pretreatment serum thyrotropin (TSH) 15.1 mU/L, interquartile range 11.5–35.3 mU/L]. HT was defined and exclusion criteria applied as previously described (15). Healthy, euthyroid control subjects (*n* = 10, 1 male), had normal ultrasound findings of the thyroid gland and were negative for thyroid autoantibodies. All participants were unrelated adults from eastern Croatia. This study protocol was reviewed and approved by the institutional ethical committee of the Osijek University Hospital, and all subjects gave written informed consent prior to the testing.

### Thyroid Function Measurement

TSH (normal range: 0.46–4.7 mIU/L, Vitros TSH Reagent Pack), free tri-iodothyronine (FT3) (1.9–5.7 pmol/L, Vitros FT3 Reagent Pack), and free T4 (FT4, 10–22 pmol/L, Vitros FT4 Reagent Pack, all from Ortho-Clinical Diagnostics, Amersham, UK) were measured in sera taken between 8 and 12 a.m., according to the manufacturer’s instructions. Maximum pretreatment TPOAb-IgG (50–125 kIU/L) was determined by ELISA kit (Anti-TPO, MileniaBiotec, Germany) calibrated against WHO reference MRC 66/387a. Hypothyroidism was defined as clinically significant when TSH was >4.7 mU/L and FT4 was <10 pmol/L, but it was considered subclinical or latent if TSH was >4.7 mU/L and FT4 was >10 pmol/L.

### Thyroid Volume Measurements

The thyroid volume was sonographically established as the sum of the volumes of the two lobes using a 10 MHz linear array transducer (Accuson X-150, Siemens, Germany). Each lobe was assumed an ovoid (π/6 × length × width × depth) as previously specified (16).

### Peripheral Blood Mononuclear Cells (PBMC) Isolation

Peripheral blood mononuclear cells were isolated from freshly collected heparinized blood samples using density gradient centrifugation on LymphoPrep (Axis Shield, Oslo, Norway) as described in details elsewhere (16). Briefly, 10 mL initial volume of whole blood was diluted with 0.9% (w/v) NaCl in 1:1 ratio, carefully layered over 20 mL LymphoPrep medium and sedimented into fractions during 20 min centrifugation at 800 × *g*. Fraction of mononuclear cells retained at the plasma/medium interface was harvested, rinsed with saline, and spun down for 10 min at 550 × *g*. Pooled cells were further rinsed in two successive wash cycles. Final PBMC pellet was resuspended with 1 mL of isolation buffer (PBS without Ca^2+^ i Mg^2+^ with 0.1% (v/v) BSA and 2 × 10^−3^ mol/dm^3^ EDTA) and cells were counted after trypan-blue staining using the Bürker–Türk counting chambers and light microscope. A minimum of 1 × 10^7^ purified PBMCs were used in the following isolation step.

### Lymphocyte Subsets Separation

Peripheral T-lymphocytes were separated from PBMCs by immunomagnetic depletion of non-CD3^+^ cells in cell suspension using DynaMag magnet and Dynabeads Untouched Human T cell Isolation Kit (Invitrogen, Paisley, UK), as described previously (3). In the first 20 min incubation step, 1 × 10^7^ PBMCs resuspended in 100 µL isolation buffer enriched with 20% (v/v) FBS, were labeled with mouse monoclonal antibodies specific for CD14, CD16, CD19, CD36, CD56, CDw123, and CD235 markers. Antibody-labeled cells and unstained CD3^+^ T-lymphocytes were rinsed, carefully mixed by tilting, and pooled down by centrifugation for 8 min at 300 *g*. Pelleted cells were resuspended in 100 µL of isolation buffer and incubated for 15 min with pre-washed magnetic beads, coated with human anti-mouse IgG antibody. Bead-bound cells adhered to the polypropylene tube walls when exposed to the DynaMag stationary magnetic field, leaving cell suspension free from B-lymphocytes, natural killer cells, monocytes, platelets, dendritic cells, granulocytes, and erythrocytes. Non-adherent CD4^+^ and CD8^+^ T cells were gently removed by pipetting to a new tube. Cell separation procedures were repeated in two successive cycles of immunomagnetic selection and washing. The final T cell collection was purified from the residual Dynabeads by placing the tube in a magnet for 2 min and then transferring the supernatant to a fresh tube.

### Total RNA Extraction

Extraction of total RNA was performed with the use of TRI REAGENT (Sigma, USA) following the single-step technique as described by Chomczinsky and Sacchi (17). The RNA integrity was examined by ethidium bromide staining in 2% agarose gel electrophoresis. Quantity and purity of RNA samples was checked by NanoDrop 1000 spectrophotometer (Thermo Fisher Scientific, USA) and verified by OD_260_/OD_280_ ratio >1.8 measurements.

### Reverse Transcription and microRNA Real-Time PCR Quantification

Sequence-specific stem loop reverse transcriptase (RT) primers and TaqMan MicroRNA Reverse Transcription Kit were used for cDNA synthesis of three candidate miRNA (miR-9-5p, miR-29a-3p, and miR-210-3p) and two reference small nuclear RNA (U6snRNA and RNU48) according to the TaqMan MicroRNA assay protocol (PE Applied Biosystems, Foster City, CA, USA). Reverse reactions were performed with 50 ng of total RNA in a 15 µL final volume comprising 1× RT buffer, 1 mM dNTP each, 3.33 U/μL MultiScribe RT, 0.25 U/μL RNase inhibitor, and 3 µL of 5× RT primer. Reaction mixture was incubated for 30 min at 16°C, 30 min at 42°C, and 5 min at 85°C, next diluted eightfold and stored in aliquotes at −20°C until use.

miRNA transcript levels were measured using the Rotor Gene 3000 instrument (Corbett Research, USA) in triplicate 15 µL quantitative real-time PCR (qRT-PCR) reactions containing 5.0 µL of cDNA, 7.5 µL of TaqMan Universal PCR Master Mix II kit, and 0.75 µL of pre-developed TaqMan miRNA expression assay (Applied Biosystems). The cycling conditions were set according to the guidelines in the manufacturer’s leaflet and the list of assays is given in Table 1.

**Table 1:** List of TaqMan assays of investigated miRNA.

Assay ID	miRBase ID	miRBase accession number	Mature miRNA sequence	Function	Target
000583	hsa-miR-9-5p	MIMAT0000441	UCUUUGGUUAUCUAGCUGUAUGA	Downregulate NF-κB, enhances IL-2 production in activated human CD4(+) T cells	BLIMP1

002112	hsa-miR-29a-3p	MIMAT0000086	UAGCACCAUCUGAAAUCGGUUA	Inhibitor of Th1 development and interferon (INF)-γ expression	INF-γ, T-bet, EOMES

000512	hsa-miR-210-3p	MIMAT0000267	CUGUGCGUGUGACAGCGGCUGA	Negative regulator of Th17 immune response	HIF1α, CTLA4, FOXP3

001006	RNU48	NR_002745	AGTGATGATGACCCCAGGTAACTC TGAGTGTGTCGCTGATGCCATCAC CGCAGCGCTCTGACC	Control miRNA assay	

001973	U6 snRNA	NR_004394	GTGCTCGCTTCGGCAGCACATATACT AAAATTGGAACGATACAGAGAAGATTA GCATGGCCCCTGCGCAAGGATGACAC GCAAATTCGTGAAGCGTTCCATATTTT	Control miRNA assay	

Ct values were collected at the fractional cycle number at which fluorescence passes fixed threshold of 0.05. The linear regression coefficient (*R*^2^) and amplification efficiency were assessed through five-point fourfold serial dilutions of the arbitrary standards prepared individually for each miRNA assay, and final values ranged between 0.994–0.998 and 92–100%, respectively. Intra-assay variability was less than 1.12%, and less than 2.01% variation was achieved between different PCR experiments. Prior to qPCR data normalization, two small nuclear RNA were tested for stability using geNorm and NormFinder algorithms. Both U6snRNA and RNU48 endogenous controls expressed the same measure of stability (M = 1.046), but U6snRNA was used for miRNA gene normalization due to the lower inter-group variability (SD ± 0.15) when compared to RNU48 (SD ± 0.22). Fold difference in miRNA expression was calculated using efficiency corrected model of 2^−ΔΔCt^ method as described by Pfaffl (18). Data are presented as relative quantity of target miRNA, normalized with respect to U6snRNA and a control group (19). A detailed description of *T-bet, FoxP3, BLIMP1*, and *HIF1*α mRNA quantification analysis, together with PCR efficiency results and stability measurements of validated housekeeping genes, can be found elsewhere (3).

### Statistical Analysis

Normality of distributions was tested by Shapiro–Wilk test. Given that data used in this study were often not normally distributed, nonparametric approaches were selected, in general. Proportions and median with interquartile range (IQR) were used for presentation of data. Exact binomial test, Wilcoxon test, and Kruskal–Wallis test with Bonferroni-corrected Dunn’s *post hoc* analysis were used for group comparisons. Correlations between paired datasets were determined by Spearman rank-test. Two-tailed *P* < 0.05 was considered significant. If not otherwise stated, statistical analyses were performed with NCSS2007 program (v07.1.20, NCSS LLC, Kaysville, UT, USA).

## Results

### HT Patients Characteristics: Biochemical and Clinical Data

Patients and healthy controls were matched for sex and age (Table 2). No difference in FT3 or TSH serum levels was observed between healthy controls, euthyroid, and L-T4 treated HT subjects; however, LT4-receiving patients required higher serum FT4 levels to attain comparable serum FT3 levels. Untreated, hypothyroid subjects demonstrated the opposite trend showing lower FT4, but higher serum TSH levels in comparison to control and LT4 substituted group. FT3 levels increased with the serum FT4 (ρ = 0.313, *P* = 0.043) concentrations in pooled sample of HT patients and controls, and declined with age in HT cases (ρ = −0.358, *P* = 0.045).

**Table 2:** Descriptive analysis of clinical and biochemical characteristics of patients and healthy controls.

	HYPO Hashimoto’s thyroiditis (HT)	SUBST HT	EU HT	CTRL	P*
Subjects (*n*)	10	10	12	10	–
Age (years)	45 (26–56)	64 (61–65)	51 (41–60)	44 (36–59)	0.069
Gender (F/M)	8/2	9/1	12/0	9/1	–
FT4 (pmol/L)	11.4 (10.2–12.5)**	15.4 (12.2–17.6)^#^	11.8 (11.3–13)	13.8 (12.4–14.6)	0.000054
FT3 (pmol/L)	2.92 (2.46–3.14)	2.83 (2.43–3.55)	2.9 (2.45–4.04)	3.38 (2.75–3.87)	0.173
TSH (mIU/L)	9.6 (5.59–13.1)	2.6 (1.19–3.25)^##^	3.11 (1.65–4.04)^##^	1.65 (0.98–2.62)^##^	<0.000001
Volume (mL)	16 (14.1–19.4)	11.1 (6.9–13.9)	14.6 (11.2–20.5)	11.9 (10.2–12.8)	0.136
TPOAb (kIU/mL)	155 (61–3,000)	260 (150–1,355)	690 (272–2,528)	Neg	–

*Kruskal–Wallis test.

**P < 0.05 vs CTRL.

*^#^P < 0.05 vs HYPO HT and EU HT*.

*^##^P < 0.05 vs HYPO HT*.

*FT4, free thyroxine; FT3, free tri-iodothyronine; TSH, thyroid stimulating hormone; TPOAb, thyroid peroxidase antibodies*.

### PB T Cells From HT Patients Display Reduced Expression of miR-29a

We analyzed the expression levels of miR-29a, miR-210, and miR-9 using RNA bank from the prior cohort as well as using published qRT-PCR data on selected target mRNAs from our previous study (3). Because no difference in expression levels of candidate miRNAs was present across different HT stages, we further analyzed HT data as one group.

**Figure 1:**
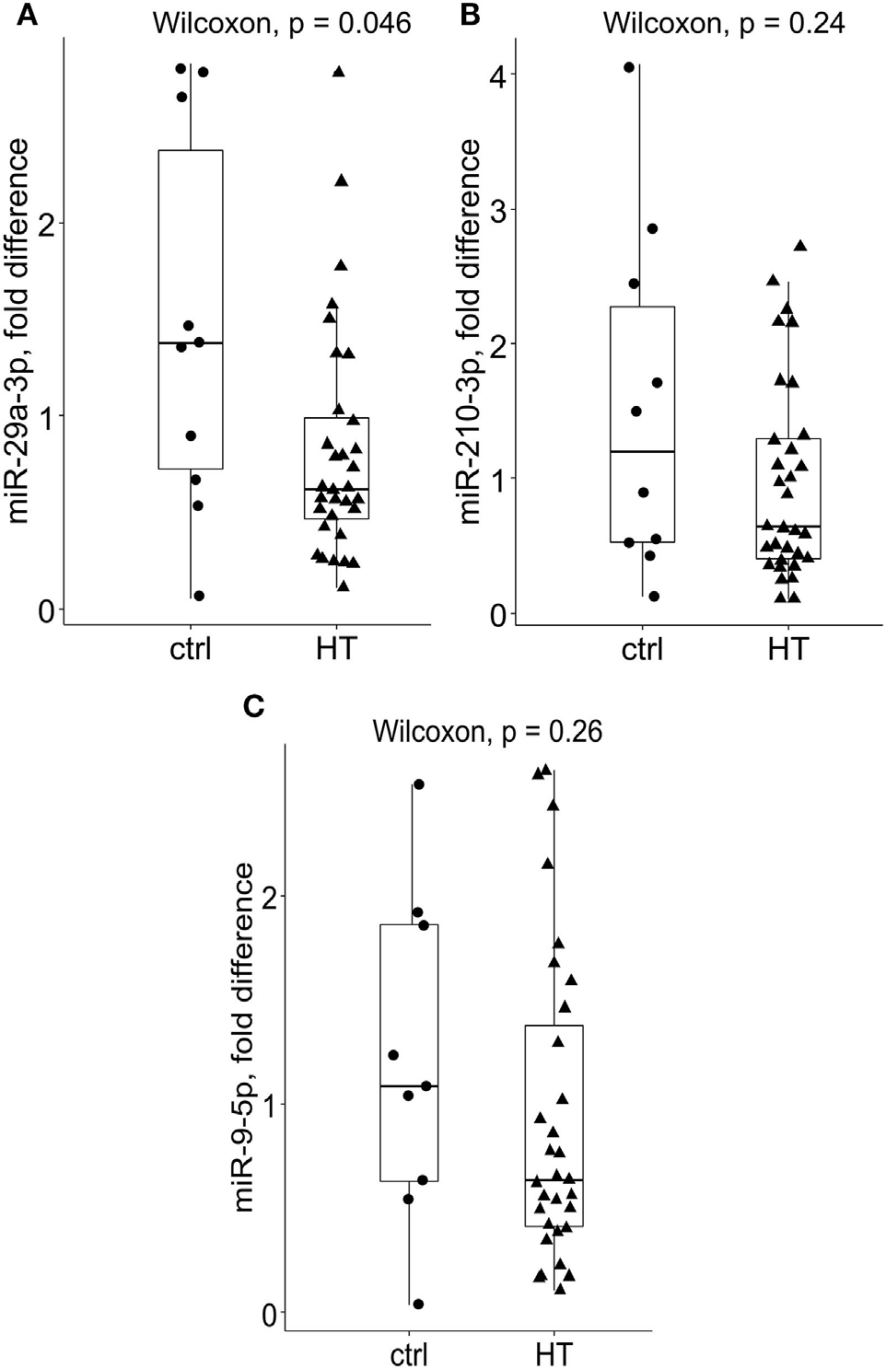
Relative expression levels of miR-29a-3p, miR-210-3p, and miR-9-5p in Hashimoto’s thyroiditis (HT) patients and healthy controls. Compared to controls, **(A)** miR-29a-3p levels were significantly reduced in bulk peripheral blood T cells of HT patients [HT (*n* = 32), *P* = 0.046, Wilcoxon test, Bonferroni–Dunn’s *post hoc* comparison]. A non-significant decrease in **(B)** miR-210-3p levels was noted in HT vs control comparisons (HT vs controls; 0.64 vs 1.2, *P* = 0.24). Conversely, no difference in **(C)** miR-9-5p expression levels was found across the studied groups. Transcriptional changes were measured by RT-qPCR and normalized against U6snRNA. Within each box, the horizontal line represents the median value, and the first and third quartiles are the ends of the box. The whiskers extend to 1.5× (interquartile range).

Subsequently, miR-29a-3p expression levels were significantly diminished in bulk PB T cells of HT patients {median fold difference 0.62 [IQR (0.44–1.01), *P* = 0.046, Wilcoxon test, Figure 1A, *n* = 32]} when compared to their healthy counterparts [1.373 (0.63–2.7), *n* = 10]. A similar, but not significant trend was observed for miR-210-3p in HT vs control comparison [HT vs ctrl; 0.64 (0.39–1.31) vs 1.2 (0.5–2.56), *P* = 0.24, Figure 1B]. Finally, no change in expression levels of miR-9-5p was seen between HT cases [0.64 (0.49–1.02) vs 1.08 (0.54–1.92), *P* = 0.26, Figure 1C] and healthy subjects.

### miRNA–mRNA Target Pairs in PB T Cells From HT Patients

**Figure 2:**
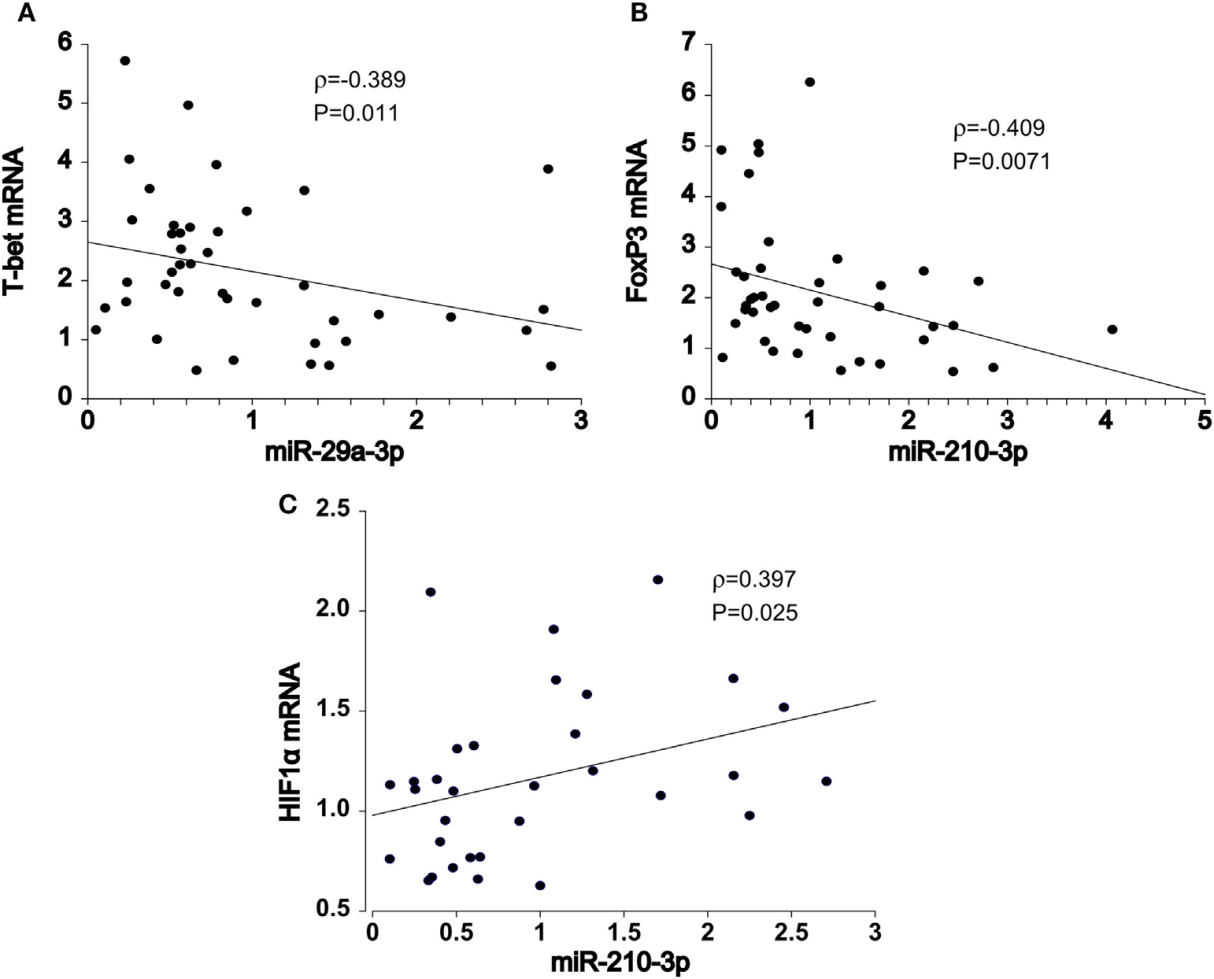
Spearman pair-wise correlation analysis of miRNA-mRNA target pairs in pooled samples (*N* = 42). Declining **(A)** miR-29a-3p expression levels was associated with increments in T-bet transcript abundance. Similar, negative relationship was also noted between **(B)** miR-210-3p and FOXP3 mRNA levels. Conversely, **(C)** miR-210-3p was positively correlated with HIF1α gene expression. Spearman rank-test, solid line: the least square estimate. ρ—Spearman correlation coefficient; global significance *P* < 0.05.

To gain further insight into the miRNA-target RNA dynamics, we integrated data from previously collected mRNA measurements with the newly obtained miRNA findings. A number of significant correlations were observed in pooled HT and control samples, majority of which was consistent with the previously reported post-transcriptional mechanisms of miR-29 (10) and miR-210 (11) action. Importantly, *T-bet* (Spearman’s correlation coefficient, ρ = −0.389, *P* = 0.011, *n* = 42, Figure 2A) mRNA levels increased with declining miR-29a-3p levels, whereas *FOXP3* transcript abundance increased with diminished miR-210-3p levels (ρ = −0.409, *P* = 0.0071, *n* = 42, Figure 2B). The associations persisted upon exclusion of control samples (data not shown); thus eliminating the confounding by case–control status. In addition, miR-210-3p seemed to be associated with *HIF1α* regulatory circuits, being positively related to *HIF1α* transcript abundance in the HT group (ρ = 0.397, *P* = 0.025, *n* = 32, Figure 2C).

### T-bet, miR-29a-3p, and miR-210-3p Transcript Levels Are Associated With the Clinical Features of HT

**Figure 3:**
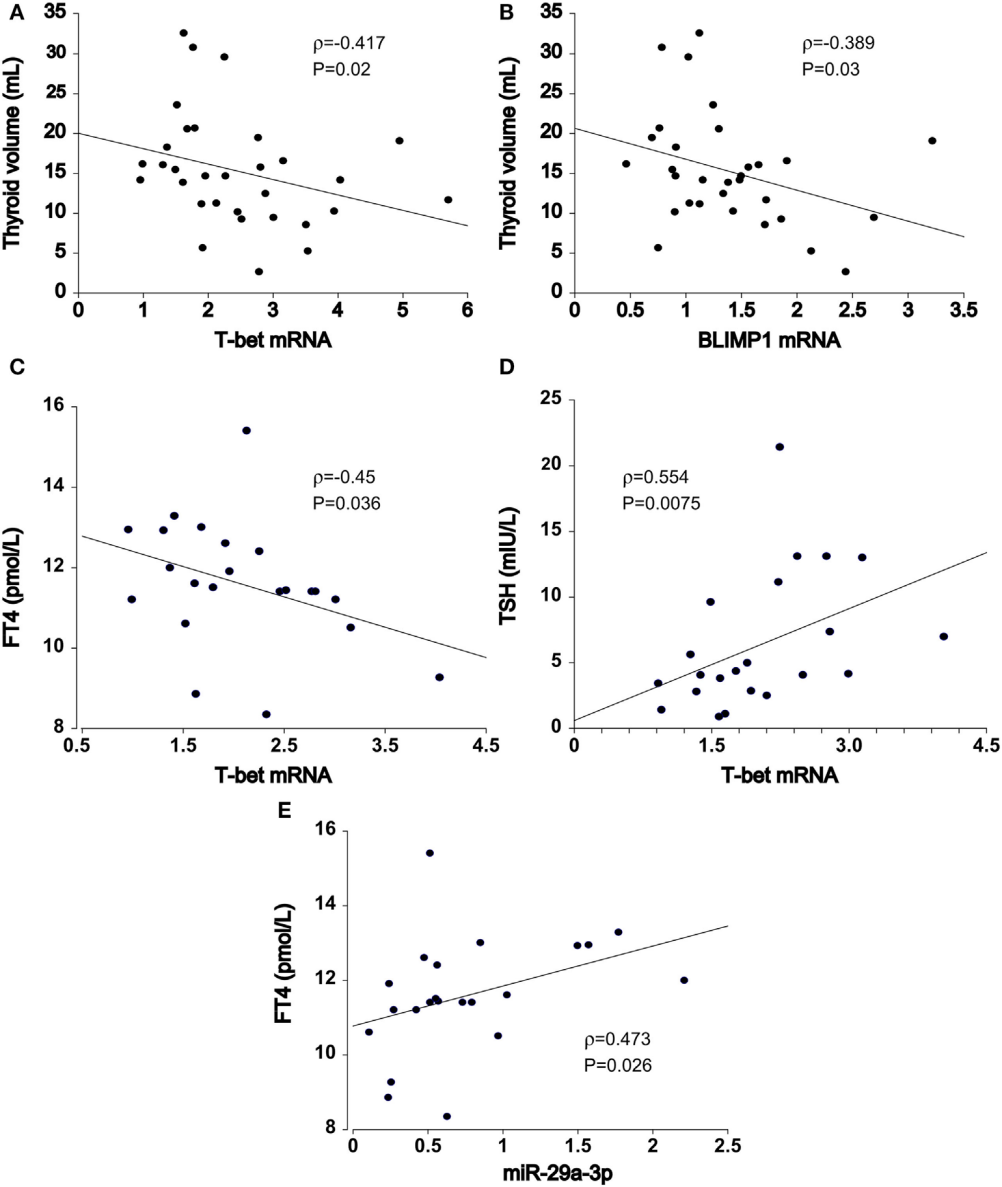
Association of T cell miRNA and mRNA transcript levels with Hashimoto’s thyroiditis (HT) clinical features. In HT patients (*n* = 32), thyroid volume was inversely related to increments in **(A)** T-bet and **(B)** BLIMP1 transcript abundance in peripheral blood T cells. In untreated HT patients (euthyroid + hypothyroid, *n* = 22), upregulated **(C)** T-bet expression was associated with declining FT4 and **(D)** increasing TSH serum levels. **(E)** miR-29-3p expression exhibited opposite, positive effects in relation to serum FT4 levels. Transcriptional changes of mRNA and miRNA were measured by quantitative real-time PCR and normalized against TBP and U6snRNA, respectively.

In HT patients, residual thyroid volume was negatively associated with increase in *T-bet* (ρ = −0.417, *P* = 0.02, *n* = 32, Figure 3A) and *BLIMP1* mRNA transcript levels (ρ = −0.389, *P* = 0.03, *n* = 32, Figure 3B) of PB T cells, particularly in euthyroid (euHT + substHT) cases [ρ(*T-bet*) = −0.483, *P* = 0.027; ρ(*BLIMP1*) = −0.558, *P* = 0.0085]. Moreover, increased *T-bet* mRNA expression was associated with reduced serum FT4 levels (ρ = −0.45, *P* = 0.036, *n* = 22, Figure 3C) and increased TSH levels (ρ = 0.554, *P* = 0.0075, Figure 3D) among the therapeutically naive patients, both spontaneously euthyroid and hypothyroid ones, independently of the individual thyroid volume [partial ρ(FT4) = −0.518, *P* = 0.016] and age [partial ρ(FT4) = −0.435, *P* = 0.049]. Conversely, miR-29a transcript levels were positively related to serum FT4 values (ρ = 0.473, *P* = 0.026) in untreated HT cases (*n* = 22, Figure 3E), whereas miR-210 levels were positively associated with FT3 levels (ρ = 0.44, *P* = 0.041). Age and thyroid volume did not alter the relation.

By univariate analysis, serum FT3 levels were associated with miR-29a-3p (ρ = 0.39, *P* = 0.0099) expression and *T-bet* (ρ = −0.439, *P* = 0.0037) transcript abundance in pooled dataset of HT cases and controls (*n* = 42). The associations persisted upon correction for FT4 levels [partial ρ(miR-29a-3p) = 0.337, *P* = 0.031, ρ(*T-bet*) = −0.39, *P* = 0.012] and age [partial ρ(miR-29a-3p) = 0.428, *P* = 0.0052, ρ(*T-bet*) = −0.374, *P* = 0.016]. No relationship was seen between miR-9 and any of the tested clinical features. Furthermore, TPOAb levels and LT4 replacement dose were not related to any miRNA or mRNA analyzed.

## Discussion

We previously observed high T-bet and FoxP3 mRNA expression in PB T cells from HT patients relative to age-matched healthy control individuals. In this study, we looked for evidence of correlated changes in miRNA-target mRNA profile by measuring transcript levels of miR-9-5p, miR-29a-3p, and miR-210-3p in an identical RNA sample set. We could now demonstrate that miR-29a-3p may be involved in the imbalance of T-bet through decreased miR-29a-3p expression in T cells of HT patients, which may play an important role in the development of HT. In parallel, negative miR-210-3p/FoxP3 and positive miR-210-3p/HIF1α circuits were also noted, possibly reflecting postranscriptional dynamics previously described in activated CD4^+^ and CD8^+^ effectors under conditions of low oxygen tension (14). Nevertheless, underlying miR-210-3p mechanisms in HT autoimmunity remain unclear, since we found no difference in miR-210-3p expression between HT and control subjects; a result supporting redundant or possibly diluted effects of miR-210-3p in mixed PB cell populations of HT patients. In contrast, downregulated miR-29-3p levels suggest a dysregulation of the miR-29/T-bet feedback loop, which may bias Th1 and Tc1 cell differentiation and contribute to chronic inflammation in HT.

The functional relevance of this altered T-bet/miR-29a-3p axis is further highlighted by finding that our untreated HT cohort with high T-bet/low miR-29a-3p expression had showed increased predisposition to develop thyroid insufficiency and primary hypothyroidism. Mechanistically, T4 and T3 synthesis are inhibited by cytokines directly on the level of thyroid epithelial cells (20): TSH-R gene is downregulated, and the expression of deiodinases (D)1 and D2, which convert T4 into the most active metabolic form, T3, is altered (21). INFγ, a prototypical T-bet/miR-29 target, is the cytokine most clearly associated with thyroid injury, especially hypothyroidism (22, 23). Increased serum interleukin (IL)-2, TNFα, and IFNγ have been reported in subjects with overt hyopothyroidism due to HT (24, 25). Hashimoto’s thyroids are enriched in intraglandular CD4^+^IFNγ^+^ and CD8^+^IFNγ^+^ T cells (26) and TPOAbs enhance the autoantigen-elicited production of IFNγ by MNCs in a dose- and Fcγ-receptor dependent manner (27). In addition, IL-12, which controls IFNγ and T-bet expression in activated T cells, is another miR-29a target (28) that has been linked to hypothyroidism in AITD (29). Alternatively, or in addition; TSH, T4, and T3, in their own turn, can play an important role in T cells and thymus and regulate their functions *via* TSH, D2, and T3 receptors. No difference, however, in T-bet/miR-29a expression was observed between LT4-treated and untreated hypothyroid HT in this study, suggesting that the correction of FT4 by hormone replacement therapy alone does not prevent T-bet/miR-29a alterations in T cells from end-stage HT. Taken together, our data are in comply with studies showing no effect of low thyroid hormone levels on T cell cytokine production (30), but instead support a role for deregulated T-bet/miR-29a immunologic background in HT state.

The important role of T-bet in controlling HT symptoms in target organs is further complemented by observation linking transcriptional heterogeneity of T-bet and BLIMP1 gene in HT PB T cells with inter-individual variations in thyroid volume, which may also contribute to the progression of thyroid insufficiency and the development of hypothyroidism. Mechanistically, both BLIMP1 and T-bet are crucial factors for the development of short-lived effector cells (SLEC) and cytotoxic T lymphocyte (CTL) (31). In this context, a co-associated TF, BLIMP1, has been considered a major TF for the generation and function of cytotoxic CD4/CD8 and effector T cells. BLIMP1 acts by promoting the binding of T-bet to the promoters of cytolytic genes in CD4^+^ T cells and is required for the cytolytic function and effector differentiation of CD4^+^ T cells and CD8^+^ CTLs, its expression peaking in antigen-experienced T cells (32). *In vivo*, inhibition of BLIMP1 reduces the production of autoantibodies and alleviates organ damage in selected autoimmune pathologies (33). By contrast, BLIMP1-deficient effector CTLs have impaired cytotoxicity, owing to their impaired expression of multiple cytotoxic molecules, including granzyme B and IFNγ, as well as the TF T-bet (34). Currently, however, the experimental link with thyroid injury yet remains to be established. Adding to the complexity of T-bet regulation, the transcriptional permissibility of Tbx21 promoter has also been described in non-Th1/Tc1 subsets, including Th17 as well as iTreg cells. Several reports thus indicate that T-bet expression is essential for generation of Th17 cells and their switch from Th17 to highly pathogenic Th1-like exTh17 cells as well (35, 36). The tight regulation of T-bet is, therefore paramount, but little understood aspect of HT.

Regarding the potential source, miRNA expression levels dynamically change between naïve, effector, and memory T cell subsets (37). A global downregulation of miRNA, including miR-29a, has been seen in effector T cells, compared to naïve T precursors, and tends to increase back in memory T cells (38). SLEC are particularly sensitive to graded expression of T-bet and BLIMP1, and the formation of terminal effector cells is largely dependent on the IL-12/T-bet axis, which drives their generation in a dose-dependent manner (31). Thus, a more precise analysis of T cell subpopulations would offer a better definition of the miR-29a activity in HT T cells.

Hashimoto’s thyroiditis is also characterized by altered composition of both FoxP3^+^ and Th17 cell compartments. HIF1α was reported to control the balance of Th17/Treg differentiation through direct transcriptional activation of RORγt; in addition, HIF1α targets FOXP3 for proteasomal degradation, thereby inhibiting Treg development (14). HIF1α acts partially by increasing the expression of miR-210 as well, which subsequently functions to inhibit both FoxP3 and HIF1α in a negative feedback loop (13, 14). Consistent with these results, we observed a modest, but significant miR-target pairing in coexpression correlations, coupled to a non-significant decrease of miR-210 expression in PB T cells from HT patients. These moderate effects might nevertheless be sufficient to contribute to the regulatory alterations behind HT, as exemplified by fine-tuning quantitative effects typically exhibited by miRNAs (39). Positive or negative correlations alone, of course, do not prove causality, since there can be a plethora of different factors involved in mRNA degradation. One must also take into account that the comparison was made using an entire PB T cell population, so it is possible that dilution and confounding by mixed cell populations, notwithstanding a wide array of post-transcriptional mechanisms, may have acted to weaken the relation specific to a high-expressing fraction of PB cell population. Finally, it is possible that the repressive effect of selected miRs is more pronounced on the translational level.

The mechanisms beyond miR-210 expression in HT thus remain unknown, and appear to play a limited role, at least under the current experimental settings. Similarly, the homogeneity of T cell populations, as well as the factors affecting the Treg purity and definition, is expected to affects the hierarchical expression of FOXP3 in HT, and therefore, the estimates of the miR-210 effect. Other, non-canonical FOXP3^+^ T cells, such as FOXP3^+^CD127^low^CD25^low^ population, are also prevalent in autoimmunity and likely to be involved in FOXP3 turnover as well (40). We also note the modest sample size and the lack of data from diseased tissues; hence, these data warrant further investigation in a validation study with a large cohort of patients, by using multiparametric labeling, longitudinal samples, and functional assays to establish the frequency and the cell type specificity of target expression. Similarly, further work will be needed to assess the roles of additional miRs, including miR-9-5p, which did not reach statistical significance in our analysis.

In conclusion, our preliminary results implicate a potentially significant role of T-bet/miR-29a-3p axis and their cellular sources in HT presentation. The results broaden our understanding of transcriptional alterations in HT, and link T-bet/miR-29a-3p pathways with the heterogeneity of clinical and laboratory manifestations among HT patients. Statistical power remains limited in small datasets; thus, more work is needed, preferably in well-powered datasets, to consolidate observed differences and clarify specific roles for the miRNA in the actual mechanisms of HT pathogenesis.

## Ethics Statement

This study was carried out in accordance with the recommendations of the institutional ethical committee of the Osijek University Hospital, with written informed consent from all subjects, and in accordance with the Declaration of Helsinki. The protocol was approved by the institutional ethical committee of the Osijek University Hospital.

## Author Contributions

Conceived and designed the experiments: MP, ST, and MŠ. Performed the experiments: ST, MŠ, LG-O, AK, and ZN. Analyzed the data: ST and MŠ. Contributed reagents/materials/analysis tools: MP, MŠ, and LG-O. Contributed to the writing of the manuscript: ST, MŠ, and MP. All the authors reviewed and approved the final manuscript.

## Conflict of Interest Statement

The authors declare that the research was conducted in the absence of any commercial or financial relationship that could be construed as a potential conflict of interest.

## References

[1] Pearce EN Farwell AP Braverman LE Thyroiditis. N Engl J Med (2003) 348(26):2646–55. 10.1056/NEJMra021194 12826640

[2] Shi Y Wang H Su Z Chen J Xue Y Wang S Differentiation imbalance of Th1/Th17 in peripheral blood mononuclear cells might contribute to pathogenesis of Hashimoto’s thyroiditis. Scand J Immunol (2010) 72(3):250–5. 10.1111/j.1365-3083.2010.02425.x 20696023

[3] Tokić S Štefanić M Glavaš-Obrovac L Jaman S Novosadová E Petrkova J The expression of T cell FOXP3 and T-bet is upregulated in severe but not euthyroid Hashimoto’s thyroiditis. Mediators Inflamm (2016) 2016:3687420. 10.1155/2016/368742 27478306 PMC4949338

[4] Figueroa-Vega N Alfonso-Pérez M Benedicto I Sánchez-Madrid F González-Amaro R Marazuela M. Increased circulating pro-inflammatory cytokines and Th17 lymphocytes in Hashimoto’s thyroiditis. J Clin Endocrinol Metab (2010) 95(2):953–62. 10.1210/jc.2009-1719 20016049

[5] Liu Y Tang X Tian J Zhu C Peng H Rui K Th17/Treg cells imbalance and GITRL profile in patients with Hashimoto’s thyroiditis. Int J Mol Sci (2014) 15(12):21674–86. 10.3390/ijms151221674 25429429 PMC4284671

[6] Li C Yuan J Zhu YF Yang XJ Wang Q Xu J Imbalance of Th17/Treg in different subtypes of autoimmune thyroid diseases. Cell Physiol Biochem (2016) 40(1–2):245–52. 10.1159/000452541 27855396

[7] Marazuela M García-López MA Figueroa-Vega N de la Fuente H Alvarado-Sánchez B Monsiváis-Urenda A Regulatory T cells in human autoimmune thyroid disease. J Clin Endocrinol Metab (2006) 91(9):3639–46. 10.1210/jc.2005-2337 16804051

[8] Xue H Yu X Ma L Song S Li Y Zhang L The possible role of CD4^+^CD25^high^Foxp3^+^/CD4^+^IL-17A^+^ cell imbalance in the autoimmunity of patients with Hashimoto thyroiditis. Endocrine (2015) 50(3):665–73. 10.1007/s12020-015-0569-y 25771887

[9] Kishore A Borucka J Petrkova J Petrek M Novel insights into miRNA in lung and heart inflammatory diseases. Med Inflamm (2014) 259131:27 10.1155/2014/259131 PMC4058468 24991086

[10] Steiner DF Thomas MF Hu JK Yang Z Babiarz JE Allen CD MicroRNA-29 regulates T-box transcription factors and interferon-γ production in helper T cells. Immunity (2011) 35(2):169–81. 10.1016/j.immuni.2011.07.009 21820330 PMC3361370

[11] Fayyad-Kazan H Rouas R Fayyad-Kazan M Badran R El Zein N Lewalle P MicroRNA profile of circulating CD4-positive regulatory T cells in human adults and impact of differentially expressed microRNAs on expression of two genes essential to their function. J Biol Chem (2012) 287(22):18584. 10.1074/jbc.M111.337154 22294691 PMC3323050

[12] Jebbawi F Fayyad-Kazan H Merimi M Lewalle P Verougstraete J-C Leo O A microRNA profile of human CD8^+^ regulatory T cells and characterization of the effects of microRNAs on Treg cell-associated genes. J Transl Med (2014) 12:218 10.1186/s12967-014-0218-x 25090912 PMC4440568

[13] Wang H Flach H Onizawa M Wei L McManus MT Weiss A Negative regulation of Hif1a expression and TH17 differentiation by hypoxia regulated miR-210. Nat Immunol (2014) 15(4):393–401. 10.1038/ni.2846 24608041 PMC3996831

[14] Dang EV Barbi J Yang HY Jinasena D Yu H Zheng Y Control of T(H)17/T(reg) balance by hypoxia-inducible factor 1. Cell (2011) 146(5):772–84. 10.1016/j.cell.2011.07.033 21871655 PMC3387678

[15] Štefanić M Papić S Suver M Glavaš-Obrovac L Karner I Association of vitamin D receptor gene 3’ variants with Hashimoto’s thyroiditis in the Croatian population. Int J Immunogenet (2008) 35(2):125–31. 10.1111/j.1744-313X.2008.00748.x 18279374

[16] Tokic S Štefanić M Karner I Glavaš-Obrovac L Altered expression of CTLA-4, CD28, VDR, and CD45 mRNA in T cells of patients with Hashimoto’s thyroiditis – a pilot study. Endokrynol Pol (2017) 68(3):274–82. 10.5603/EP.2017.0010 28660994

[17] Chomczynski P Sacchi N. Single-step method of RNA isolation by acid guanidinium thiocyanate-phenol-chloroform extraction. Anal Biochem (1987) 162:156–9. 10.1006/abio.1987.9999 2440339

[18] Pfaffl MW. A new mathematical model for relative quantification in real-time RT-PCR. Nucleic Acids Res (2001) 29(9):2002–7. 10.1093/nar/29.9.e45 11328886 PMC55695

[19] Novosadova E Chabronova A Kolek V Petrek M Navratilova Z The serum expression of selected miRNAs in pulmonary sarcoidosis with/without Löfgren’s syndrome. Med Inflamm (2016) 2016:1246129 10.1155/2016/1246129 PMC5165170 28050119

[20] Jakobs TC Mentrup B Schmutzler C Dreher I Köhrle J. Proinflammatory cytokines inhibit the expression and function of human type I 5’-deiodinase in HepG2 hepatocarcinoma cells. Eur J Endocrinol (2002) 146(4):559–66. 10.1530/eje.0.1460559 11916626

[21] Xu G Tu W Qin S. The relationship between deiodinase activity and inflammatory responses under the stimulation of uremic toxins. J Transl Med (2014) 12:239. 10.1186/s12967-014-0239-5 25174507 PMC4155120

[22] Gérard AC Boucquey M van den Hove MF Colin IM Expression of TPO and ThOXs in human thyrocytes is downregulated by IL-1-alpha/IFN-gamma, an effect partially mediated by nitric oxide. Am J Physiol Endocrinol Metab (2006) 291(2):E242–53. 10.1152/ajpendo.00439.2005 16478776

[23] Marique L Van Regemorter V Gérard AC Craps J Senou M Marbaix E The expression of dual oxidase, thyroid peroxidase, and caveolin-1 differs according to the type of immune response (TH1/TH2) involved in thyroid autoimmune disorders. J Clin Endocrinol Metab (2014) 99(5):1722–32. 10.1210/jc.2013-3469 24476075

[24] Mazziotti G Sorvillo F Naclerio C Farzati A Cioffi M Perna R Type-1 response in peripheral CD4 and CD8 T cells from patients with Hashimoto’s thyroiditis. Eur J Endocrinol (2003) 148:383–8. 10.1530/eje.0.1480383 12656657

[25] Karanikas G Schuetz M Wahl K Paul M Kontur S Pietschmann P Relation of anti-TPO autoantibody titre and T-lymphocyte cytokine production patterns in Hashimoto’s thyroiditis. Clin Endocrinol (Oxf) (2005) 63(2):191–6. 10.1111/j.1365-2265.2005.02324.x 16060913

[26] Bossowski A Harasymczuk J Moniuszko A Bossowska A Hilczer M Ratomski K Cytometric evaluation of intracellular IFN-g and IL-4 levels in thyroid follicular cells from patients with autoimmune thyroid diseases. Thyroid Res (2011) 4:13 10.1186/1756-6614-4-13 21943174 PMC3189885

[27] Liu Y You R Yu N Gong Y Qu C Zhang Y Increased proportions of Tc17 cells and NK cells may be risk factors for disease progression in Hashimoto’s thyroiditis. Int Immunopharmacol (2016) 40:332–8. 10.1016/j.intimp.2016.09.016 27668571

[28] Brain O Owens BM Pichulik T Allan P Khatamzas E Leslie A The intracellular sensor NOD2 induces microRNA-29 expression in human dendritic cells to limit IL-23 release. Immunity (2013) 39(3):521–36. 10.1016/j.immuni.2013.08.035 24054330

[29] Mikoś H Mikoś M Obara-Moszyńska M Niedziela M. The role of the immune system and cytokines involved in the pathogenesis of autoimmune thyroid disease (AITD). Endokrynol Pol (2014) 65(2):150–5. 10.5603/EP.2014.0021 24802739

[30] Kiyici S Gul OO Baskan EB Hacioglu S Budak F Erturk E Effect of levothyroxine treatment on clinical symptoms and serum cytokine levels in euthyroid patients with chronic idiopathic urticaria and thyroid autoimmunity. Clin Exp Dermatol (2010) 35:603–7. 10.1111/j.1365-2230.2009.03642.x 19874329

[31] Xin A Masson F Liao Y Preston S Guan T Gloury R A molecular threshold for effector CD8+ T cell differentiation controlled by transcription factors Blimp-1 and T-bet. Nat Immunol (2016) 17:422–32. 10.1038/ni.3410 26950239 PMC5779087

[32] Kallies A Xin A Belz GT Nutt SL Blimp-1 transcription factor is required for the differentiation of effector CD8+ T cells and memory responses. Immunity (2009) 31:283–95. 10.1016/j.immuni.2009.06.021 19664942

[33] Luo J Niu X Zhang M Zhang K Chen M Deng S. Inhibition of B lymphocyte-induced maturation protein-1 reduces the production of autoantibody and alleviates symptoms of systemic lupus erythematosus. Autoimmunity (2015) 48(2):80–6. 10.3109/08916934.2014.976627 25347333 PMC4389764

[34] Boulet S Daudelin J-F Labrecque N. IL-2 induction of Blimp-1 is a key in vivo signal for CD8+ short-lived effector T cell differentiation. J Immunol (2014) 193(4):1847–54. 10.4049/jimmunol.1302365 25015830

[35] Lazarevic V Chen X Shim J-H Hwang ES Jang E Bolm AN T-bet represses TH17 differentiation by preventing Runx1-mediated activation of the gene encoding RORγt. Nat Immunol (2016) 12:96–104. 10.1038/ni.1969 PMC3077962 21151104

[36] Wang Y Godec J Ben-Aissa K Cui K Zhao K Pucsek AB The transcription factors T-bet and Runx are required for the ontogeny of pathogenic interferon-γ-producing T helper 17 cells. Immunity (2014) 40(3):355–66. 10.1016/j.immuni.2014.01.002 24530058 PMC3965587

[37] Teteloshvili N Smigielska-Czepiel K Kroesen BJ Brouwer E Kluiver J Boots AM T-cell activation induces dynamic changes in miRNA expression patterns in CD4 and CD8 T-cell subsets. Microrna (2015) 4(2):117–22. 10.2174/2211536604666150819194636 26290349

[38] Haoquan W Neilson JR Kumar P Manocha M Shankar P Sharp PA miRNA profiling of naïve, effector and memory CD8 T cells. PLoS One (2007) 2(10):e1020. 10.1371/journal.pone.0001020 17925868 PMC2000354

[39] Lai X Wolkenhauer O Vera J. Understanding microRNA-mediated gene regulatory networks through mathematical modelling. Nucleic Acids Res (2016) 44(13):6019–35. 10.1093/nar/gkw550 27317695 PMC5291278

[40] Ferreira RC Simons HZ Thompson WS Rainbow DB Yang X Cutler AJ Cells with Treg-specific FOXP3 demethylation but low CD25 are prevalent 2 in autoimmunity. J Autoimmun (2017) 84:75–86. 10.1101/134692 28747257 PMC5656572

